# Quality of life and mental health of women who had cardiac disease in pregnancy and postpartum

**DOI:** 10.1186/s12884-022-05123-x

**Published:** 2022-10-28

**Authors:** Jane Hutchens, Jane Frawley, Elizabeth A. Sullivan

**Affiliations:** 1 School of Public Health, Faculty of Health, University of Technology Sydney, Ultimo, NSW Australia; 2 Faculty of Health and Medicine, University of Newcastle, Callaghan, Australia

**Keywords:** Cardiac, Pregnancy, Postpartum, Quality of life, Mental health

## Abstract

**Purpose:**

Cardiac disease is a leading cause of maternal morbidity and mortality yet there is limited research on women’s experiences and quality of life (QoL) outcomes. The aim of this study is to explore the general and health-related QoL (HRQoL) and mental health outcomes for women who have experienced cardiac disease in pregnancy and the first 12 months postpartum (CDPP).

**Methods:**

This exploratory descriptive study recruited 43 women with acquired, genetic and congenital CDPP. Patient reported outcomes measures (PROMs) used were: WHOQoL-Bref, a Kansas City Cardiac Questionnaire (KCCQ), the Depression, Anxiety and Stress Scales-21 (DASS-21), the Cardiac Anxiety Questionnaire (CAQ) plus newly developed questions.

**Results:**

Women reported low health satisfaction (51.7/100), physical health (55.2/100) and low HRQoL (63.1/100). Women had clinically significant scores for depression (24%), anxiety (22%) and stress (19.5%) (DASS-21) and 44.5% scored at least moderate anxiety on the CAQ. Most women (83.7%) were advised to avoid pregnancy which 88.9% found “upsetting” to “devastating”; 10.0% were offered counselling. Most women were concerned about reduced longevity (88.1%), offspring developing a cardiac condition (73.8%), and the limitations on enjoyment of life (57.1%). Women missed medical appointments due to cost (25.03%) and difficulty arranging childcare (45.5%).

**Conclusion:**

The majority of women reported inadequate information and counselling support, with women with CDPP having sustained impaired QoL and mental health outcomes. The new and modified questions relating to mothering and children reflected the primacy of mothering to women’s identity and needs.

**Supplementary information:**

The online version contains supplementary material available at 10.1186/s12884-022-05123-x.

## Background

Cardiac disease complicates 1–4% of pregnancies and is a lead cause of maternal morbidity and mortality in low- and high-income countries [1]. Prevalence data for cardiac disease in the first 12 months postpartum is less defined, however overall prevalence in pregnancy and postpartum is increasing [2, 3]. Cardiac disease in pregnancy and postpartum (CDPP) encompasses a disparate range of acquired, congenital and genetic conditions. Women with CDPP have a higher risk of poor quality of life (QoL) and mental health outcomes due to the concurrence of multiple factors relating to particular cardiac factors and personal characteristics. Cardiac factors include: the association of cardiac disease with poorer mental health [4, 5], prognostic uncertainty, cardiac disease and lower QoL [6], and the specific challenges experienced by adults with congenital heart disease [7] and younger people with cardiac disease in general [8]. Personal characteristics that reduce QoL include: the presence of pre-existing and perinatal mental health conditions [9], the effects of maternal near-miss events [10], and the experience of mothering with a chronic illness [11, 12]. These associations have mental health and QoL implications however, they are yet to be fully examined.

Disease-specific PROM tools are optimal [13] and while there are multiple tools available for various cardiac diseases [14], and for pregnancy and postpartum [15], at present there are no validated PROMs specific to individual cardiac diseases in pregnancy and postpartum. A few studies have adapted existing tools to fill this gap [16].

Patient reported outcomes measures use standardised, validated patient-completed questionnaires to quantify patient’s perceptions of their health and functional status, independent of interpretation by healthcare professionals (HCPs) or researchers [17, 18]. Patient reported outcomes are essential in understanding and improving clinical and broader QoL outcomes and are integral to person-centred care and shared decision-making [19]. Patient-reported anxiety, depression, perceived health, QoL and distress is an independent predictor of subsequent hospital readmission and costs, morbidity and mortality, and knowledge of these factors can aid in risk assessment and resource development and allocation [19, 20].

Of the limited data available on patient-reported outcomes for women with CDPP most prevalent were findings of poorer mental health and lower QoL outcomes [16]. It has also been reported that anxiety (generalised and cardiac-specific) and reduced quality of life persist up to 10 years after women’s CDPP [21]. Further, women with CDPP are at an increased risk of being diagnosed with a major mental health disorder [22] as a consequence of their illness and score almost twice as high for anxiety and depression as non-pregnant women and men with the same cardiac condition [23, 24].

This exploratory study sought to understand the QoL and mental health for women who had cardiac disease in pregnancy and the first year postpartum in Australia. Understanding these women’s experiences will guide further research and provide important data for clinicians and healthcare services to improve the lives of women with CDPP.

## Methods

### Study protocol and participants

This exploratory descriptive study was developed in response to a previous qualitative study by the authors that identified ongoing reduced QoL and mental health challenges for women who had CDPP [25]. Data for the present study were collected from an anonymous online survey using the Qualtrics Software (Nov 2021) and analysed using IBM SPSS Statistics for Windows, Version 28.0. Ethics approval was granted by the University of Technology Sydney’s *Human Research Ethics Committee.*

Participants were recruited via Facebook and cardiac organisations from August to November 2021. Participation was voluntary and did not attract any financial benefit. A detailed description of the study was provided, and consent was confirmed by commencing the survey. Inclusion criteria were Australian residents or citizens who were living in Australia when they had cardiac disease during any pregnancy or in the first 12 months postpartum and who gave birth to one or more babies beyond 20 weeks’ gestation or 400gm or greater birthweight; were 18 years of age and older and who had sufficient English language skills to understand this information and complete the survey. All cardiac diseases were included excluding a primary diagnosis of hypertension or preeclampsia.

### Measures

The following survey instruments were used to capture a breadth of data on this under-researched population.

### WHOQoL-Bref

The WHOQoL-Bref was chosen as the generic QoL instrument because its theoretical construct is based on a broad concept of QoL and health that is not limited to biomedical aspects. The WHOQOL-Bref is a well-established validated tool, used in studies on a variety of physical conditions such as CHD [26], postpartum [27], rheumatoid arthritis [28], depression [29], posttraumatic stress disorder [30] and anxiety and stress [31].

The Australian WHOQoL-Bref questionnaire is an abridged version of the WHOQoL-100 [32]. It contains 2 global questions (overall QoL and health satisfaction) and 24 questions divided into four domains: Domain 1 physical health (7 items), Domain 2 psychological health (6 items), Domain 3 social relationships (3 items) and Domain 4 environment (8 items). For this study we excluded Q26 “How often do you have negative feelings such as blue mood, despair, anxiety, depression?” due to repetition with other instruments in the study, leaving 5 items in Domain 2 (psychological health). Each item is rated on a 5‑point Likert scale. The raw domain scores are scaled in a positive direction and transformed to a 0-100 scale for comparison with WHOQoL-100. The two global questions are scaled in a positive direction with a score range of 1–5. Higher scores for both the domains and the global questions indicate higher QoL. Domain scores were calculated according to the Guide and transformed to be out of 100 [33]. Cronbach’s Alpha was used to demonstrate internal consistency of the WHOQoL-Bref scale (0.921).

### Modified kansas city cardiac questionnaire (KCCQ)

The KCCQ is a health related QoL (HRQoL) instrument for individuals with heart failure that quantifies the domains of physical limitations, symptoms, self-efficacy and knowledge, social interference, and QoL [34, 35]. The KCCQ focus is on the presence, severity, and impact of heart failure symptoms on functionality. Item responses are coded sequentially (e.g., “not at all satisfied” to “completely satisfied”) from worst to best status. We have incorporated adaptations to the KCCQ by Koutrolou-Sotiropoulou, Lima (16) relating to work status, counselling and future pregnancies. These items were coded on a scale, and some also included free text sections.

The cardiac HRQoL was augmented by including modified questions relevant to mothers and younger patients such as concerns regarding longevity, their children, finances, sex and access to care from an recent study on women with peripartum cardiomyopathy (PPCM) [21]. Our study included women with a diversity of cardiac diagnoses so the above cardiac HRQoL tools were modified to be applicable for all participants. Novel questions were developed to distinguish between information and counselling provided at time of diagnosis, rate the quality of information and communication, determine if counselling was provided in response to advice to avoid having further children and what impact that advice had on participants, and frequency of missing medical appointments due to cost. These new questions were developed after our qualitative study which found a lack of recognition of the mental health impact of CDPP in both the short and long term, and subsequent lack of mental health referral or support provided [25] . Affirmative responses to scaled questions were consolidated to a single positive response (e.g., a little, moderate, very) for the purpose of analysis. Cronbach’s Alpha was used to demonstrate internal consistency of the KCCQ scale (0.919).

Both the WHOQoL-Bref and the KCCQ include measures more accurately described as measures of disability, or limitation, such as ability to walk around the block, and these are possibly more applicable to an older population [36, 37]. Most of these types of questions were retained to facilitate comparison of results. Additional questions introduced by Koutrolou-Sotiropoulou, Lima (16) measured clinical outcomes such as ejection fraction and medication use rather patient-reported outcomes therefore they were not included in this study as they were not patient centred.

### Depression, anxiety, and stress scales-21 (DASS-21)

The DASS-21 is a validated self-report scale with three subscales of depression, anxiety, and stress [38, 39]. The DASS-21 was chosen as the general mental health instrument as it differentiates between depression, anxiety and stress, is short and simple to complete, and is a key tool used in research, general practice and mental health services in Australia [40].

The depression subscale items focus on low mood, hopelessness, low self-esteem, ability to feel pleasure and inertia. The anxiety subscale items focus on physiological arousal, situational anxiety, and feelings of panic and fear. The stress subscale items focus on difficulty in relaxing, impatience, irritability, and chronic non-specific arousal. Each subscale has 7 items, all answered on a Likert scale from 0 (“Did not apply to me at all’’) to 3 (“Applied to me very much, or most of the time’’) [39, 41]. The scores for the total DASS-21 and for each subscale are summed, with low scores reflecting better mental health. Cronbach’s Alpha was used to demonstrate Internal consistency of the DASS-21 scale (0.908).

### Cardiac anxiety questionnaire (CAQ)

The CAQ was used as a specific assessment of cardiac anxiety, which is apprehension and fear related to cardiac-related stimuli and sensations [42]. The CAQ has 18 items with 3 subscales of fear (8 items), avoidance (5 items), and heart-focused attention (5 items). Each item is rated on a 5-point rating scale from 0 (“never”) to 4 (“always”). The total score is calculated as the mean of all items. Subscale scores are calculated as the mean of the relative frequency ratings for each of the items in each subscale. There are no validated clinical cut-off scores however according to the grading of the items, a higher total and subscale scores indicate greater cardiac-related anxiety [42, 43]. Cronbach’s Alpha was used to demonstrate Internal consistency of the CAQ scale (0.852).

### Analysis

Data were summarised using descriptive statistics and included frequencies and percentages for categorical data, with ranges, means, and standard deviations for continuous data. Validated instruments were scored according to the requirements of each instrument. Data were considered based on time since CDPP (< 5 years or > 5years) and combined category of disease (acquired or combined congenital and genetic heart disease).

## Results

### Participant characteristics

A total of 43 women completed the survey. Women reported a range of acquired, congenital and genetic conditions, including; cardiomyopathies, rhythm disorders, coronary artery dissection and myocardial infarction, structural anomalies and valvular conditions (see *Appendix A*). Timing of diagnosis of the cardiac condition ranged from birth through to 12 months postpartum. Mean age at the time of first CDPP was 31.39 years (19–39), mean time since the first pregnancy with CDPP was 4.9 years. See *Appendix B* for participant characteristics.

### The WHOQoL-Bref

The transformed score out of 100 for overall QoL was 80.8 +/-21.7 and health satisfaction and 51.7 +/- 25.2. WHOQoL-Bref results are outlined in Table 1.

**Table 1:** WhOQol-Bref scores

	**Score**	**SD +/-**
Overall QoL	80.8	21.7
Health satisfaction	51.7	25.2
Physical health	55.2	11.9
Psychological health	64.9	17.1
Social relationships	63.0	22.1
Environment	72.2	21.7

Health satisfaction was scored as “very dissatisfied/dissatisfied” for 37.5% of participants whose CDPP was less than 5 years ago, and 35% of those whose CDPP was more than 5 years ago. While significance is difficult to determine due to the sample size, it is worth noting the continued dissatisfaction with their health for 35% of participants who experienced CDPP more than five years prior to the survey. .

### KANSAS and additional questions

WHOQoL-Bref scores QoL as a single question and the KCCQ QoL is a composite score of 3 questions; both are scored out of 100. The mean WHOQoL score for overall QoL was 84.6, compared to the mean HRQoL score from the modified KCCQ of 63.1. Domain results of the modified KCCQ are outlined in Table 2.

**Table 2:** Modified KCCQ scores

Quality of life	63.1
Physical limitation	72.6
Self-efficacy	75.5
Symptoms	73.2
Social limitations	79.1

**Fig. 1:**
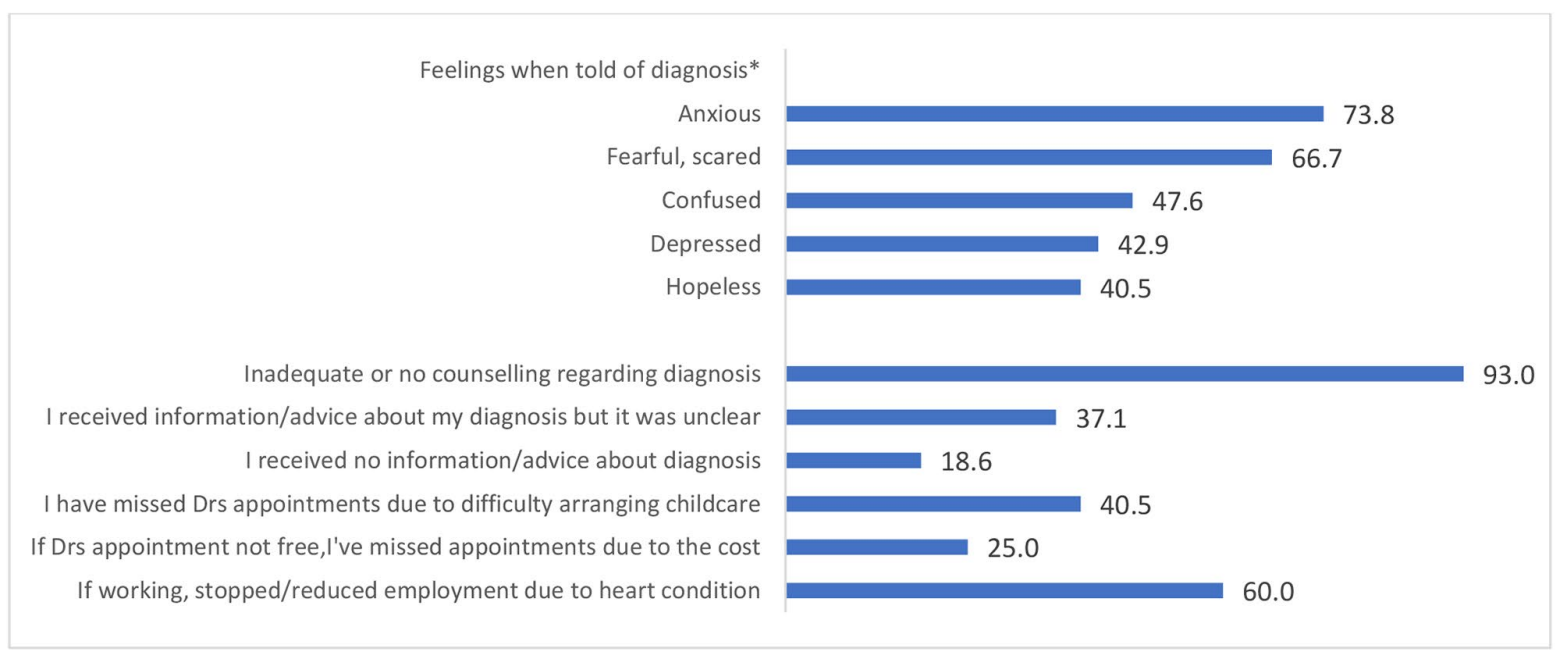
Communication, access and support. (* Or was old enough to understand diagnosis)

**Fig. 2:**
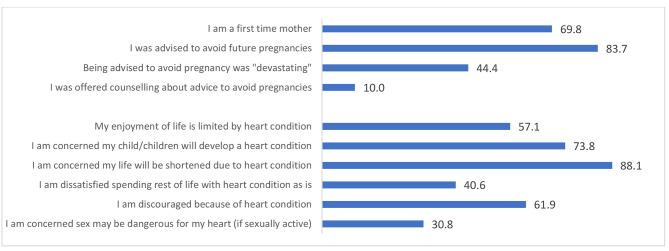
Key concerns

Key results for the combined cardiac HRQoL questions are shown in Figs. 1 and 2. The majority of women were advised to avoid future pregnancies which 88.9% scored as “upsetting” through to “devastating” with 10.0% offered counselling. Concern about reduced life expectancy due to heart condition was high (total 88.1%) and was not fully moderated by time, with 40% of women who had CDPP in the past 18 months and 48.4% who had CDPP 18 months or longer ago scoring “very concerned”. Many women worried about their children developing a cardiac condition (73.8%). Women were at times unable to attend medical appointments due to cost (25.0%) and difficulty arranging childcare (40.5%). A summary of key results of the combined cardiac HRQoL are summarised in Figs. 1 and 2.

### DASS-21

Depression was scored as “moderate”, “severe” or “extremely severe” for 24% of participants, anxiety 22% and stress 19.5%. Women diagnosed during pregnancy had the highest scores for depression (50.0%) and stress (33.4%) and women diagnosed before pregnancy scored highest for anxiety (28.5%). By category, women with acquired heart disease scored highest for depression (30.4%) and stress (26.1%), and women with congenital heart disease scored highest for anxiety (36.4%). Core Dass-21 results are provided in Tables 3 and 4. It is noted that it is difficult to determine significance with small sample size.

**Table 3:** DASSs-21 scores

	**Mean**	**Std deviation**
Depression	4.29	3.82
Anxiety	3.41	2.89
Stress	6.27	3.83
Total DASS	13.98	9.20

**Table 4:** DASS-21 scores for category of disease and time since CDPP

	**Acquired**		**CHD/genetic**				**< 5yrs since diagnosis**		**> 5yrs since diagnosis**			
**Anxiety**	**n**	**%**	**n**	**%**	**p =**	**Total**	**n**	**%**	**n**	**%**	**p =**	**Total**
Yes	5	22%	4	22%	0.970	9	6	25%	3	19%	0.643	9
No	18	78%	14	78%		32	18	75%	13	81%		31
	23		18			41	24		16			40
Depression												
Yes	7	30%	3	14%	0.308	10	7	30%	3	19%	0.456	10
No	16	70%	15	86%		31	17	70%	13	81%		30
	23		18			41	24		16			40
Stress												
Yes	6	26%	2	11%	0.230	8	6	25%	2	12%	0.330	8
No	17	74%	16	89%		33	18	75%	14	88%		32
	23		18			41	24		16			40

### Cardiac anxiety questionnaire

The total CAQ score was 34.6 +/- 10.22. Of the subscales, fear (1.86) and avoidance (1.67) are lowest and heart-focused attention (1.87) is highest in those diagnosed before pregnancy. By disease category fear was highest for women with acquired heart disease (2.10), avoidance highest in genetic (2.09) and lower for CHD (1.47) and heart-focused attention highest in CHD (1.84). The highest scoring individual item was “I pay attention to my heartbeat” (71.4%). Core CAQ results are provided in Table 5.

**Table 5:** Cardiac anxiety questionnaire scores

	**Mean**	**Std. deviation**
Fear	2.00	0.612
Avoidance	2.02	0.877
Heart focused attention	1.71	0.729
Overall	1.92	0.568

## Discussion

There is a paucity of research into the QoL and mental health of women who have had CDPP, despite the increasing prevalence of CDPP and its associated morbidity, mortality. In this exploratory study we describe the QoL and mental health of women who had CDPP in Australia. Our novel findings include: (1) the primacy of issues relating to motherhood, mothering and children, (2) the need for and lack of mental health support, (3) a substantial difference in the generic QoL compared to the HRQoL, (4) poor outcomes for HRQoL, health satisfaction and mental health, (5) poor QoL and mental health outcomes did not necessarily improve with time since diagnosis.

Our study found that the mean for overall QoL in the WHOQoL-Bref was comparable to Australian norms, however health satisfaction was lower for women with CDPP [32]. The WHOQoL-Bref domain scores were markedly lower than norms for similarly aged females, in particular for the domains of physical health, (55.2 vs. 80.3), psychological (64.9 vs. 73.6) and social relationship (63.0 vs. 74.8) [44].

Women scored themselves lower for QoL on the modified KCCQ HRQoL as opposed to the generic WHOQoL-Bref. This may be due to specific questions related to cardiac function on the HRQoL scale, suggesting disease-specific measures may be more sensitive and better able to pick up subtle changes and impacts. Higher levels of other social and environmental determinants of health that influence QoL such as housing security, and access to healthcare (most people in Australia can access universal healthcare) may affect this score or the HRQoL may be more sensitive to the issues pertinent to participants as it a specific cardiac QoL tool. The majority of research using the KCCQ is with older individuals with heart failure and include clinical and treatment outcomes, precluding comparison with our cohort of childbearing women [45]. Individuals with low QoL and HRQoL have an increased for further cardiac events and increased mortality risk [46, 47].

The scores for QoL and the domains did not necessarily improve substantially over time, reflecting the chronicity of CDPP, and that support and services need to continue beyond standard perinatal timeframes of six weeks postpartum. Further, this suggests the need for longitudinal studies of women who experience CDPP. Nearly three-quarters of women surveyed were anxious about their children developing a heart condition; this likely reflects understanding of the condition and the level of information and genetic counselling provided.

Clinically significant levels of anxiety and depression were scored for both the DASS-21 and the CAQ. DASS-21 scores for the total and three subscales of depression, anxiety and stress were higher than all-age Australian norms (13.98 vs. 8.30, 4.29 vs. 2.57, 3.41 vs. 1.74 and 6.27 vs. 3.99 respectively) [40]. Participants were twice as likely to score at least moderate anxiety on the CAQ (45%) compared to the DASS-21 (22%) which may reflect sensitivity and or specificity of heart-related anxiety compared to general anxiety. The total and domain scores for the CAQ were comparable to Rosman’s study of women with peripartum cardiomyopathy [21], and notably higher than studies on cardiac patients in general [42, 48] and people presenting to the emergency department with non-cardiac chest pain [49]. The scores were decidedly higher than norms for women of a similar age range who didn’t have cardiac disease [50]. The CAQ subscale of avoidance may indicate heart-related anxiety, or it may reflect appropriate self-management and following medical advice, and this is worth further examination. Questions relating to interactions with HCPs and access to healthcare are also noteworthy, reflecting anxiety about their health and management by health professionals. Importantly, cardiac anxiety, depression and other mental health conditions are associated with increased risk for further cardiac events and mortality [51], in particular, in women [52]. These responses suggest a need for improved information and knowledge sharing, skill development and counselling [53].

Our results are consistent with related cardiac, mental health and maternal health research. Being female is associated with an increased risk for anxiety and depression in a variety of cardiac conditions and is associated with increased health utilisation, morbidity and mortality [23, 54, 55]. Further, individuals living with conditions associated with sudden cardiac death, such as Long QT Syndrome are at increased risk of anxiety, fear, depression and lower QoL [56–58]. Adults with implantable cardioverter defibrillators (ICDs) are reported to have higher anxiety in general and specifically shock-related anxiety and this is associated with lower sexual functioning scores, another important aspect of QoL [59]. Individuals living with genetic cardiac conditions are at risk of lower HRQoL, and higher levels of anxiety and depression compared with population norms [57]. In addition, experiencing depression, including postpartum depression is associated with lower QoL, physical satisfaction and mental health scores [60]. QoL and anxiety and depression may be modifiable with improved communication, psychological interventions and support, increased knowledge and genetic counselling as indicated [57, 58].

In an environment of limited research on CDPP, PROs offer an opportunity to enhance researcher and clinical knowledge, clinical outcomes, and QoL for women. Using PROMs can lead to better symptom recognition which is especially relevant as cardiac disease is under-recognised and under-reported in females [61–63]. PROMs enable patients to describe issues or respond to questions about issues that were not usually discussed, assisting them to highlight unmet needs [14, 36, 64–66]. The use of PROMs may prompt different communication approaches and content, leading to greater exchange of information, improved relationships, greater referrals, and co-ordination of care, as well as increased person-centred care [14, 66–68]. Finally, being involved in providing feedback through PROs is associated with improved psychological outcomes, HRQoL and patient satisfaction [61, 67, 68].

The results of this study indicate all the above characteristics of PROMs are desirable and may address the negative aspects of their PROs; however, this is dependent on the attributes of the PROM. A content comparison of 34 cardiac disease PROMs found a preponderance of PROMS related to physical and emotional functions, with no explicit mention of issues related to parenting or mothering which was paramount for our participants [13]. A systematic review of research on cardiac disease in pregnancy found 94% reported only on clinical or adverse effects and none included PROs on life impact and functioning [69]. However, pregnant women with cardiac disease want QoL and mental health included as PROs and these should be incorporate alongside the more clinically based outcomes [69, 70]. The CDPP-specific questions in our survey were the items that had the strongest responses, highlighting their importance to participants. It is imperative that women with CDPP are involved in future PROM design to capture these issues as well as topics including contraception counselling and use, sexual (dys)function, pregnancy and postpartum, and parenting and to be patient-centred [71–75].

## Strengths and limitations

This study is the first to our knowledge that includes a diversity of cardiac conditions across the three categories of acquired, congenital and genetic heart disease, and the first to include women who were diagnosed up to 12 months postpartum. This extended timeframe is particularly relevant for pregnancy-associated spontaneous coronary artery dissection and peripartum cardiomyopathy where late cases are diagnosed up to a year postpartum. In addition, we included a diversity of PROMs to capture a broad range of data and developed new questions that revealed important outcomes for women with CDPP. Participants had their first pregnancy affected by CDPP on average 5 years prior to completing the survey and this provides a useful longer-term perspective, and the reported prevalence of negative HRQoL and mental health outcomes highlights the persistence of issues related to CDPP. In addition, surveying patients close to a health event can lead to a distortion of results with survival gratefulness and optimism being overstated, and the effects of attempting to re-enter life, resume normal functions, work and social interactions, and in the case of CDPP, embark on mothering with a cardiac condition, may not yet be fully realised and thus unable to be captured at this early stage [76].

There are also clear limitations to this descriptive study. Recruitment was limited to those using Facebook or on mailing lists of cardiac organisations and registries, and recruited numbers were below what was required to perform further statistical analysis and to be considered representative. We did not record disease severity which is associated with degree of QoL impairment [77]. Additional topics that would be valuable to examine but were not included are those relating to breastfeeding and having difficulty due to chest wall surgery or being advised to cease breastfeeding due to cardiac medications, the impact on sexual relationships, and the specific issues associated with treatments (e.g., ICDs). Further, it is noted that some women’s rating of experiences relates to healthcare episodes many years ago, and may be affected by recall bias or services may have changed since that time. These limitations notwithstanding, there are important strengths in understanding women’s experiences, and these provide weight for arguing for a better healthcare experience informed by evidence-based research.

## Recommendations

- Women centred research co-designed with consumers to inform person-centred care and improve QoL and mental health outcomes.
- Co-designed research into the mental health and QoL impacts on partners and children of women with CDPP.
- Co-development of patient-centred PROMs for CDPP (beyond clinical maternal and cardiac outcomes); including mothering, relationships, breastfeeding, fertility.
- Longitudinal studies with multiple timepoints, with adequate representation from each category and severity of cardiac disease.
- Funded cardiac research to include in all analytic plans sex disaggregated data and for women of reproductive age pregnancy/postpartum status.

## Conclusion

This study found women with CDPP had reduced QoL and mental health outcomes which was not necessarily ameliorated over time. This had an additive effect of increased risk of poorer cardiac outcomes and increased health service utilisation. Some differences were noted based on category of disease and timing of diagnosis of CDPP, however longitudinal studies are required to confirm and expand on this. Women need increased support, information, and opportunities to engage as active participants in their healthcare, noting the centrality of being a mother to their experience, identity, and needs.

While in recent years more women are included as participants in research, issues around mothers and mothering remain largely invisible in CDPP research. This has resulted in issues that are relevant to women such as having further children, breastfeeding, contraception, the impact on their ability to mother in the way they want to, fear for the children’s cardiac health and their own ability to survive long enough to raise their children not being acknowledged or investigated. This could be addressed in routine clinical care through engaging women with CDPP to develop comprehensive and sensitive disease-specific PROMs. These can be integrated into clinical management and provide an opportunity to characterise women’s health and QoL in the longer-term, evaluate the impact of health interventions, and improve outcomes.

## Electronic supplementary material

Below is the link to the electronic supplementary material.

Supplementary Material 1

## Data Availability

All data generated or analysed during this study are included in this published article.

## Abbreviations

CAQ: Cardiac Anxiety Questionnaire
CDPP: Cardiac disease in pregnancy and the first year postpartum
DASS-21: Depression, Anxiety and Stress Scales-21
HCP: Healthcare professional
HRQoL: Health-related quality of life
KCCQ: Kansas City Cardiac Questionnaire
PROM: Patient reported outcomes measure
QoL: Quality of life
