# Supplementary material for: Quality of life and mental health of women who had cardiac disease in pregnancy and postpartum
Source: BMC Pregnancy Childbirth. 2022 Oct 28;22:797. doi: 10.1186/s12884-022-05123-x (PMC9617394; doi:10.1186/s12884-022-05123-x)
Supplement: Supplementary file 1 — Supplementary Material 1 [file 12884_2022_5123_MOESM1_ESM.docx]

**Quality of life and mental health of women who had cardiac disease in pregnancy and postpartum**

**Appendix A**

**Diagnoses**

Several women had multiple conditions, diagnosed simultaneously or at separate times. Conditions included:

- Arrhythmogenic mitral valve prolapse
- Arrhythmogenic Right Ventricular Dysplasia/Cardiomyopathy (ARVD)
- Atrial septal defect (ASD)
- Cardiac arrest
- Coarctation and stenosis of the aorta
- Congenitally corrected transposition of the great arteries (ccTGA)
- Dextrocardia
- Fontan circulation
- Hypertrophic Cardiomyopathy
- Idiopathic cardiomyopathy
- Leaky valve
- Long QT Syndrome
- Peripartum cardiomyopathy (PPCM)
- Pregnancy related spontaneous coronary artery dissection (PSCAD)
- Prolapsed pulmonary valve
- Pulmonary stenosis
- Supraventricular tachycardia
- Transposition of the great vessels
- Tetralogy of fallot
- Transposition of great arteries
- Truncus interrupted aortic arch
- Ventricular septal defect (VSD)

**Appendix B**

| **Participant characteristics** | |  |  |
| --- | --- | --- | --- |
|  |  | Frequency | Percent |
| Age cardiac disease diagnosed (years | 0-5 | 9 | 20.9 |
|  | 6-18 | 1 | 2.3 |
|  | 19-30 | 13 | 30.2 |
|  | 31 | 20 | 46.5 |
|  |  |  |  |
| Age of first CDPP (years) | Missing | 2 | 4.7 |
|  | Up to 25 | 3 | 7.0 |
|  | 26 – 30 | 18 | 41.9 |
|  | 31 - 36 | 12 | 27.9 |
|  | 37 or older | 8 | 18.6 |
|  |  |  |  |
| Pregnancy for first CDPP | 1st pregnancy | 30 | 69.8 |
|  | 2nd pregnancy | 7 | 16.3 |
|  | 3rd or later pregnancy | 6 | 14.0 |
|  |  |  |  |
| Number of pregnancies with CDPP | One | 30 | 69.8 |
|  | Two | 6 | 14.0 |
|  | Three or more | 7 | 16.3 |
|  |  |  |  |
| Timing of diagnosis | Before pregnancy | 14 | 32.6 |
|  | During pregnancy | 6 | 14.0 |
|  | Postpartum | 23 | 53.5 |
|  |  |  |  |
| Category of cardiac disease | Acquired | 25 | 58.1 |
|  | Congenital | 11 | 25.6 |
|  | Genetic | 7 | 16.3 |
